# Editorial: Utilizing advanced genomics and biochemical tools to strengthen crop adaptation for biotic and abiotic stresses

**DOI:** 10.3389/fpls.2025.1766137

**Published:** 2026-01-26

**Authors:** Gurjeet Singh, Santosh Gudi, Simardeep Kaur, Diaa Abd El Moneim

**Affiliations:** 1 Texas A&M University, AgriLife Research Center, Beaumont, TX, United States; 2 North Dakota State University, Fargo, ND, United States; 3 Division of Crop Science, ICAR- Research Complex for North Eastern Hill Region, Meghalaya, Umiam, India; 4 Department of Plant Sciences, North Dakota State University, Fargo, ND, United States; 5 Department of Plant Production (Genetic Branch), Faculty of Environmental Agricultural Sciences, Arish University, El-Arish, Egypt

**Keywords:** climate change, genomics tools, multi-omics approaches, biochemical pathways, biotic stress resistance, abiotic stress tolerance, sustainability

## Overview

Global agriculture faces unprecedented challenges such as emerging pests and diseases, and increasing incidence of abiotic stresses such as drought, salinity, waterlogging, and heat, threatening crop productivity and food security. The Research Topic “*Utilizing Advanced Genomics and Biochemical Tools to Strengthen Crop Adaptation for Biotic and Abiotic Stresses,”* published in *Frontiers in Plant Science*, comprises 11 scientific articles (ten original research and one review) contributed by 86 researchers worldwide. This Research Topic showcases the integration of advanced genomics, molecular breeding, and biochemical approaches to develop climate-resilient crops. Using high-throughput sequencing, genome-wide association studies (GWAS), QTL mapping, transcriptome and metabolomic analyses, key genomic regions and metabolic pathways linked to stress tolerance were identified in wheat, maize, soybean, sugarcane, pigeonpea, *Brassica napus*, and *Salvia miltiorrhiza*. Significant contributions include the identification of stripe rust resistance loci in wheat, drought-tolerant clones in sugarcane, and pod borer tolerance genetic loci in pigeonpea. Machine learning approaches prioritized stress-associated genes in maize, while comparative transcriptomics in *S. miltiorrhiza* revealed MAPK cascade roles in pathogen response and metabolite accumulation. Soybean studies identified genes and pathways that enhance tolerance to drought, salinity, and biotic stresses, including the CAM/CML gene family, which confers dual viral and fungal resistance. Additional insights include drought-responsive microRNAs in wheat and waterlogging resilience in *B. napus*. A review of genotyping in climate-smart breeding emphasizes the use of integrative tools to accelerate genetic improvement. Therefore, this RT demonstrates how integrating genomics, biochemical profiling, and computational biology can accelerate the development of high-yielding, stress-resilient cultivars as well as helps in contributing to sustainable, climate-smart agriculture.

## Published articles and summaries

1.*Identification of new genetic resources for drought tolerance-related traits from the world Erianthus germplasm collection* (Ramanathan et al.)

A diverse panel of 223 *Erianthus* germplasm accessions from seven countries were evaluated under field-imposed drought stress to identify donor clones for sugarcane improvement. Physiological screening and multi-year phenotyping enabled the development of a 91-clone drought response association panel. GWAS using 1,044 high-quality SNPs identified 43 QTNs (quantitative trait nucleotides) associated with drought-adaptive traits. Candidate genes analysis from the identified QTL regions revealed the set of genes involved in stress perception and signaling, including TOR2, TMK1, potassium, and nitrate transporters. These drought-tolerant clones, QTNs, and gene targets provide valuable resources for developing drought-resilient sugarcane cultivars through marker-assisted breeding.

2.*Genome wide association studies (GWAS) for identification of stripe rust resistance loci in diverse wheat genotypes* (Tanwar et al.)

Stripe rust, caused by *Puccinia striiformis f.* sp. *tritici* (Pst), remains a major threat of wheat productivity in North India due to rapidly evolving virulent races. To identify durable resistance, a GWAS was conducted on 652 elite wheat genotypes using 1,938 DArTseq SNPs markers and phenotypic data from four locations. The analysis revealed 27 significant genomic regions associated with stripe rust resistance, including loci on chromosomes 2B, 6A, and 6B. Candidate gene analysis from the QTL region identified defense-related genes such as NBLRR, F-box, LRR, and kinase families. These loci provide strong targets for developing user-friendly markers and accelerating breeding of rust-resistant wheat varieties.

3.*Genome-wide characterization and stress-responsive expression analysis of the cinnamoyl-CoA reductase gene family in soybean* (Li et al.)

A genome-wide analysis identified 15 CCR (cinnamoyl-CoA reductase) members in soybean across 12 chromosomes using comparative genomics, domain validation, and phylogenetics. Promoter, motif, and synteny analyses revealed diversified regulatory elements and evolutionary expansion. Transcriptome profiling under four abiotic stresses (salt, alkaline, drought, and osmotic) showed strong root-specific and stress-responsive expression, with *GmCCR1*, *GmCCR4*, *GmCCR7*, *GmCCR8*, and *GmCCR15* significantly upregulated. *GmCCR4* exhibited the most robust induction, thus indicating a key role in lignin-mediated stress adaptation. These CCR members provide molecular targets for marker-assisted breeding and genetic engineering to develop salt-alkali-tolerant, climate-resilient soybean varieties, supporting sustainable production in degraded soils.

4.*Dissecting genomic regions and candidate genes for pod borer resistance and component traits in pigeonpea minicore collection* (Moghiya et al.)

A panel of 146 pigeonpea minicore accessions, along with resistant and susceptible checks, were evaluated over three field seasons for pod borer resistance and related traits. Whole-genome resequencing generated 499,980 SNPs, enabling multi-locus GWAS using SUPER and FarmCPU models. Analysis identified 14 significant MTAs across five chromosomes, linked to key candidate genes including *carboxylesterase 15*, *microtubule-associated protein 5*, and *FAR1-related sequence*. Four accessions (ICP 10503, ICP 655, ICP 9691, and ICP 9655) showed moderate resistance in pod bioassays. These MTAs, genes, and resistant lines provide strong resources for marker-assisted breeding of pod borer–resistant pigeonpea varieties.

5.*Comprehensively characterize the soybean CAM/CML gene family, as it provides resistance against both the soybean mosaic virus and *Cercospora sojina* pathogens* (Zhang et al.)

This study identifies key regulators of broad-spectrum disease resistance through a genome-wide characterization of 113 CAM/CML genes (11 *GmCAMs* and 102 *GmCMLs*) in soybean. Phylogenetic, structural, and cis-regulatory analyses revealed 14 evolutionary groups enriched with hormone and stress-responsive elements. Expression profiling under soybean mosaic virus and *Cercospora sojina* infections at the V3 stage, five susceptible and five tolerant genotypes were identified 19 CAM/CML genes responsive to both pathogens, with *GmCAM4*, *GmCML23*, and *GmCML47* strongly associated with resistance. These findings provide a valuable resource for understanding calcium-mediated defense mechanisms and offer promising targets for molecular breeding and genome editing to develop multi-disease-resistant soybean cultivars.

6.*Predictive prioritization of genes significantly associated with biotic and abiotic stresses in maize using machine learning algorithms* (Pradhan et al.)

This study integrated 39,756 RNA-seq datasets from maize under diverse biotic and abiotic stresses to identify candidate genes for stress resilience. Using seven machine learning-based models and WGCNA, 235 top-ranked genes were prioritized, including hub genes such as *Zm00001eb176680* (*bZIP* transcription factor 68), *Zm00001eb176940* (glycine-rich structural protein 2), and *Zm00001eb179190* (*ALDH11*). Promoter analysis revealed enrichment for abscisic acid and antioxidant-responsive elements, suggesting regulatory roles in stress adaptation. These findings provide a comprehensive resource for understanding maize stress responses and offer key molecular targets for functional validation, genome editing, and molecular breeding to develop multi-stress-resilient maize cultivars for sustainable agriculture.

7.*Novel and conserved drought-responsive microRNAs expression analysis in root tissues of wheat (Triticum asetivum L.) at reproductive stage* (Sharma et al.)

This study presents a comprehensive analysis of drought-responsive microRNAs (miRNAs) in drought-tolerant (NI5439) and susceptible (WL711) wheat genotypes under control and drought stress conditions at the booting stage. A total of 364 miRNAs (306 known and 58 novel) were identified from four sRNA libraries. 18 miRNAs showed significant changes in expression after stress treatment, with 2,300 predicted target genes involved in signal transduction, epigenetic regulation, and development. Ten novel miRNAs were validated via qRT-PCR, confirming genotype-specific responses. These findings expand the catalog of drought-responsive miRNAs, providing molecular targets for functional genomics and offering future opportunities for genetic engineering and breeding drought-resilient wheat cultivars.

8.*Comparative transcriptomic analysis and genome-wide identification provide insights into the potential role of fungal-responsive MAPK cascade genes in tanshinone accumulation in Salvia miltiorrohiza* (Abozeid et al.)

This study provides the first genome-wide characterization of the MAPK gene family in *S. miltiorrhiza* and its role in tanshinone biosynthesis under fungal elicitation. A total of 17 *SmMAPKs*, 7 *SmMAPKKs*, and 22 *SmMAPKKKs* genes distributed across nine chromosomes were identified and were grouped into TEY and TDY subfamilies. Transcriptome and HPLC analyses revealed that yeast extract and *Aspergillus niger* significantly enhanced tanshinone accumulation, particularly cryptotanshinone and dihydrotanshinone. *SmMAPK4* and *SmMAPKK5* showed strong positive correlations with tanshinone content, suggesting a regulatory role. These findings provide molecular targets for genetic engineering and fungal elicitor-based strategies to enhance tanshinone production, paving the way for improved medicinal applications.

9.*Deciphering salt tolerance mechanisms in synthetic hexaploid and bread wheat under humic acid application: physiological and genetic perspectives* (Alghabari and Shah)

This study evaluated the effect of humic acid (HA) on salt tolerance in four synthetic hexaploid (SH) and three bread wheat (BW) genotypes for physiological, biochemical, and genetic traits. HA enhanced chlorophyll content (33.3-100%), photosynthesis (31.2-50%), and antioxidant enzyme activities (SOD, POD, CAT), while reduced Na^+^/K^+^ ratio (33.3-50%), proline (20-28.5%), and glycine betaine (42.8-77.7%) under salt stress. Salinity-associated genes (*TaNHX1*, *TaHKT1*,*4*, *TaAKT1*, *TaPRX2A*, *TaSOD*, and *TaCAT1*) were upregulated, whereas *TaP5CS* was downregulated, in SH wheat lines, indicating superior tolerance. SH genotypes can serve as a bridge to transfer salt tolerance traits into wheat breeding programs. Future studies should conduct field evaluations across diverse soils and climates to optimize HA application and assess long-term impacts on wheat salt resilience.

10.*Genome-wide identification of PDX and expression analysis under waterlogging stress exhibit stronger waterlogging tolerance in transgenic Brassica napus plants overexpressing the BnaPDX1.3 gene compared to wild-type plants* (Yao et al.)

This study characterizes the PDX gene family in *B. napus* variety G218, revealing 13 PDX genes with high evolutionary conservation and roles in waterlogging stress response. Functional analysis of *BnaPDX1.3* overexpressing plants demonstrated enhanced vitamin B6 synthesis, stronger antioxidant enzyme activity, stable ROS homeostasis, and improved biomass under waterlogging compared to wild-type plants. The results highlight *BnaPDX1.3* as a key regulator of waterlogging tolerance *B. napus*. These findings provide a foundation for molecular breeding and genetic engineering strategies to develop waterlogging-tolerant *B. napus* cultivars. Author concluded that future research could explore regulatory networks, cross-stress tolerance, and field-level validation to accelerate climate-resilient crop development.

11.*The importance of genotyping within the climate-smart plant breeding value chain integrative tools for genetic enhancement programs* (Garcia-Oliveira et al.)

This review highlights the key role of genotyping and integrative tools in climate-smart plant breeding to enhance crop resilience and productivity. Modern approaches, including genome editing, mutation breeding, multi-omics, microRNAs, and digital technologies, enable precise targeting of loci controlling complex traits and support real-time decision-making. Climate-smart plant breeding innovations are relevant for smallholder farmers across diverse agro-climatic zones, helping crops adapt to climate variability, nutritional demands, and consumer preferences. The article also emphasizes responsible adoption, addressing genetic erosion, biodiversity, and intellectual property concerns. Overall, integrating these tools offers a holistic strategy for sustainable, climate-resilient agricultural systems.

